# Zinc-modified Calcium Silicate Coatings Promote Osteogenic Differentiation through TGF-β/Smad Pathway and Osseointegration in Osteopenic Rabbits

**DOI:** 10.1038/s41598-017-03661-5

**Published:** 2017-06-13

**Authors:** Jiangming Yu, Lizhang Xu, Kai Li, Ning Xie, Yanhai Xi, Yang Wang, Xuebin Zheng, Xiongsheng Chen, Meiyan Wang, Xiaojian Ye

**Affiliations:** 1 Department of Orthopaedics, Changzheng Hospital of Second Military Medical University, Shanghai, 200003 China; 2 Key Laboratory of Inorganic Coating Materials, Shanghai Institute of Ceramics, Chinese Academy of Sciences, Shanghai, 200050 China; 3 Shanghai Key Laboratory of Regulatory Biology, Institute of Biomedical Sciences and School of Life Sciences, East China Normal University, Shanghai, 200241 China; 4 National Engineering Research Center for Nanotechnology, Shanghai, 200241 China

## Abstract

Surface-modified metal implants incorporating different ions have been employed in the biomedical field as bioactive dental implants with good osseointegration properties. However, the molecular mechanism through which surface coatings exert the biological activity is not fully understood, and the effects have been difficult to achieve, especially in the osteopenic bone. In this study, We examined the effect of zinc-modified calcium silicate coatings with two different Zn contents to induce osteogenic differentiation of rat bone marrow-derived pericytes (BM-PCs) and osteogenetic efficiency in ovariectomised rabbits. Ti-6Al-4V with zinc-modified calcium silicate coatings not only enhanced proliferation but also promoted osteogenic differentiation and mineralized matrix deposition of rat BM-PCs as the zinc content and culture time increased *in vitro*. The associated molecular mechanisms were investigated by Q-PCR and Western blotting, revealing that TGF-β/Smad signaling pathway plays a direct and significant role in regulating BM-PCs osteoblastic differentiation on Zn-modified coatings. Furthermore, *in vivo* results that revealed Zn-modified calcium silicate coatings significantly promoted new bone formation around the implant surface in osteopenic rabbits as the Zn content and exposure time increased. Therefore, Zn-modified calcium silicate coatings can improve implant osseointegration in the condition of osteopenia, which may be beneficial for patients suffering from osteoporosis-related fractures.

## Introduction

Osteoporosis is a chronic bone disease impacting the general population and characterized by compromised bone strength predisposing to increased fracture risk. Metal implants are used to support prosthetic devices including dental implants in the biomedical field. However, these metal implants are susceptible to corrosion, leading to implant loosening, wear and poor loading^1^, especially in osteoporotic patients, which could cause a reduction in the support ability of an implant due to decreased bone mass^2^. To overcome these issues, various strategies have been investigated. Coating the surface with bioactive components appears to be a promising way to improve the osseointegration of metal implants^3, 4^. Calcium phosphate, including hydroxyapatite (HA)^5–9^, coatings applied on titanium implants enhance the bone integration of metal implants compared to uncoated implants. However, the biological and mechanical fixation differed substantially for coated implants in osteoporotic bones and normal bones^10–14^. Hence, there is a need to modify these implants so that they can form strong bonds with the host tissue, especially in clinically challenging scenarios, such as osteoporosis due to low bone mass density and strength.

In recent years, titanium surfaces have been modified by incorporating different ions to improve the mechanical fixation of implants to bone. Zinc is a structural constituent of some proteins, including enzymes belonging to cellular signaling pathways and transcription factors, that stimulates osteoblastic cell proliferation, differentiation and mineralization^15^. Supplementation with zinc may be important in the prevention of osteoporosis^16^. Some studies have shown that Zn, Mg and Sr ion implants stimulated osteoblastic cell proliferation and had positive effects on osseointegration in both healthy or pathological bone^17–19^. Moreover, another reports have suggested that incorporation of dopant ions into bio-glass, Ca-P, and Ca-Si ceramics with certain dosage positively affected the cellular responses^20, 21^. Although scientifically promising, the molecular mechanism underlying the effects of the ion-doped bioactive coatings on bone-forming cells is not fully understood.

Bone marrow-derived pericytes (BM-PCs) as a population of progenitor cells are able to differentiate into several types of cells, including endothelial cells and osteocytic cells^22, 23^, which are used in promising cellular therapies in regenerative medicine. In the present study, We used BM pericytes to investigate the effect of Ca_2_ZnSi_2_O_7_ coatings with different Zn/Ca ratios on proliferation and osteogenic differentiation *in vitro* and the associated molecular mechanisms. Moreover, we evaluated the osteogenic potential of these coatings in osteopenic rabbits. We found that Ca_2_ZnSi_2_O_7_ coatings with higher Zn content significantly increased the cell proliferation, osteogenic differentiation genes expression and the mineralized matrix deposition. In addition, Ca_2_ZnSi_2_O_7_ coatings activated the TGF-β/Smad pathway during the osteogenic differentiation of BM-PCs. Furthermore, *in vivo* tests revealed that Zn-modified calcium silicate coatings had a significant influence on the new bone formation around the implant surface as the Zn-Ca ratio and the exposure time increased in osteopenic rabbits. The results could provide valuable information for future studies aimed to exploit positive ion-modified coatings for bone tissue engineering, especially for clinically challenging diseases such as osteoporosis.

## Results

### Effects of Various Coatings on Cell Proliferation and Cytotoxicity

**Figure 1:**
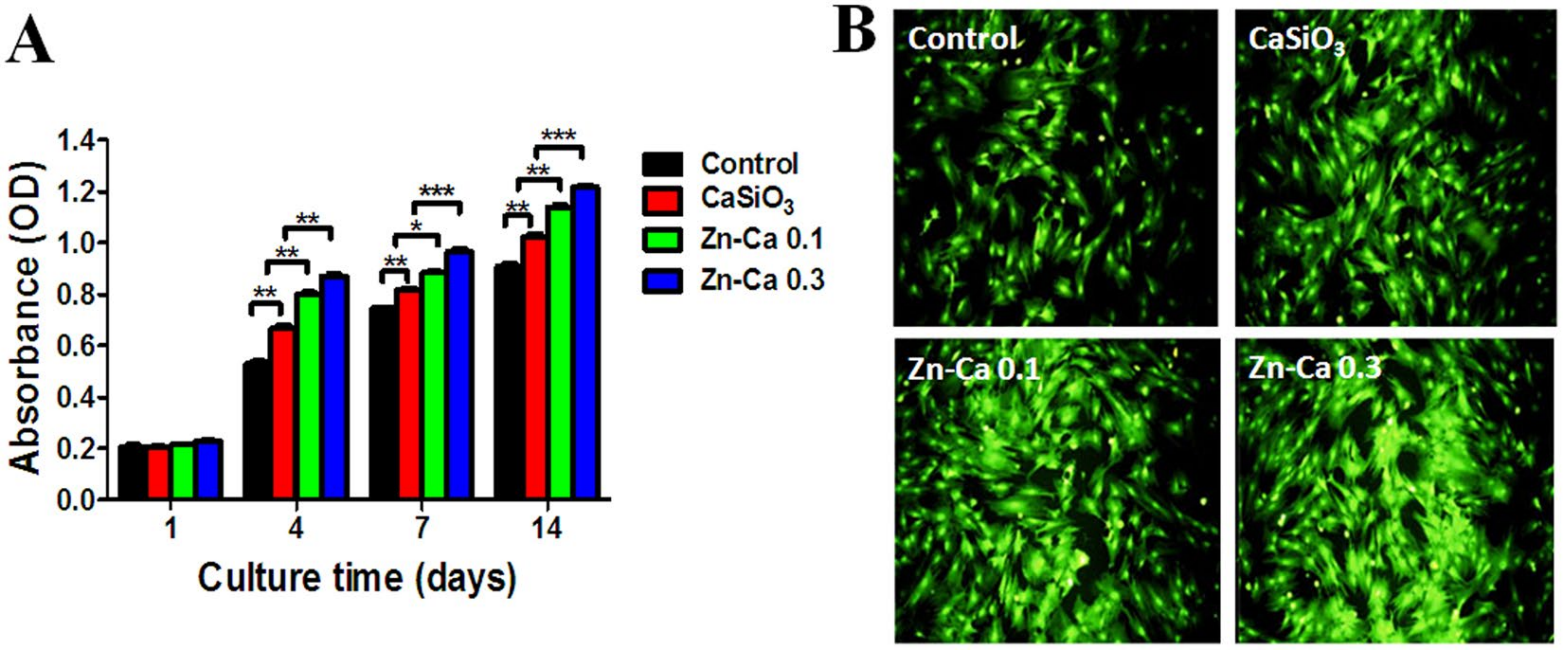
(**A**) BM-PCs proliferation on the Ti-6Al-4V (control) and the CaSiO_3_, Zn-Ca 0.1 and Zn-Ca 0.3 coating surfaces for 1, 4, 7 and 14 days as measured by a CCK-8 assay. (**B**) Cell cytotoxicity of BM-PCs cultured on different coatings at 48 h. Data are presented as the mean ± SD (n = 6). *p < 0.05, **p < 0.01, ***p < 0.001.

BM-PCs were seeded and grown on Ti-6Al-4V (control), CaSiO_3_, and Zn-Ca 0.1 and Zn-Ca 0.3 coatings. After 1, 4, 7 and 14 days, cell proliferation was determined using the Cell Counting Kit-8 (CCK-8) assay (Fig. 1A). Cell proliferation increased with the culture time and Zn content, but no difference was observed on the initial day of culture. After 4, 7 and 14 days of culture, more BM-PCs were found on the Zn-Ca 0.3 coating than on the Zn-Ca 0.1 and CaSiO_3_ coatings and the Ti-6Al-4V control. In addition, the cell cytotoxicity on the various coatings at 48 h was evaluated by a live/dead-staining assay. As shown in Fig. 1B, most BM-PCs were stained green in color and had almost no dead red-stained cells. This result was similar to that of the proliferation assay. Among all the samples, those with the Zn-Ca 0.3 coating had the largest number of living cells. Together, It has been well demonstrated that the release of suitable concentration of zinc could stimulate cell proliferation *in vitro*.

### Effect of Zinc-modified Coatings on Osteogenic Marker Expression

**Figure 2:**
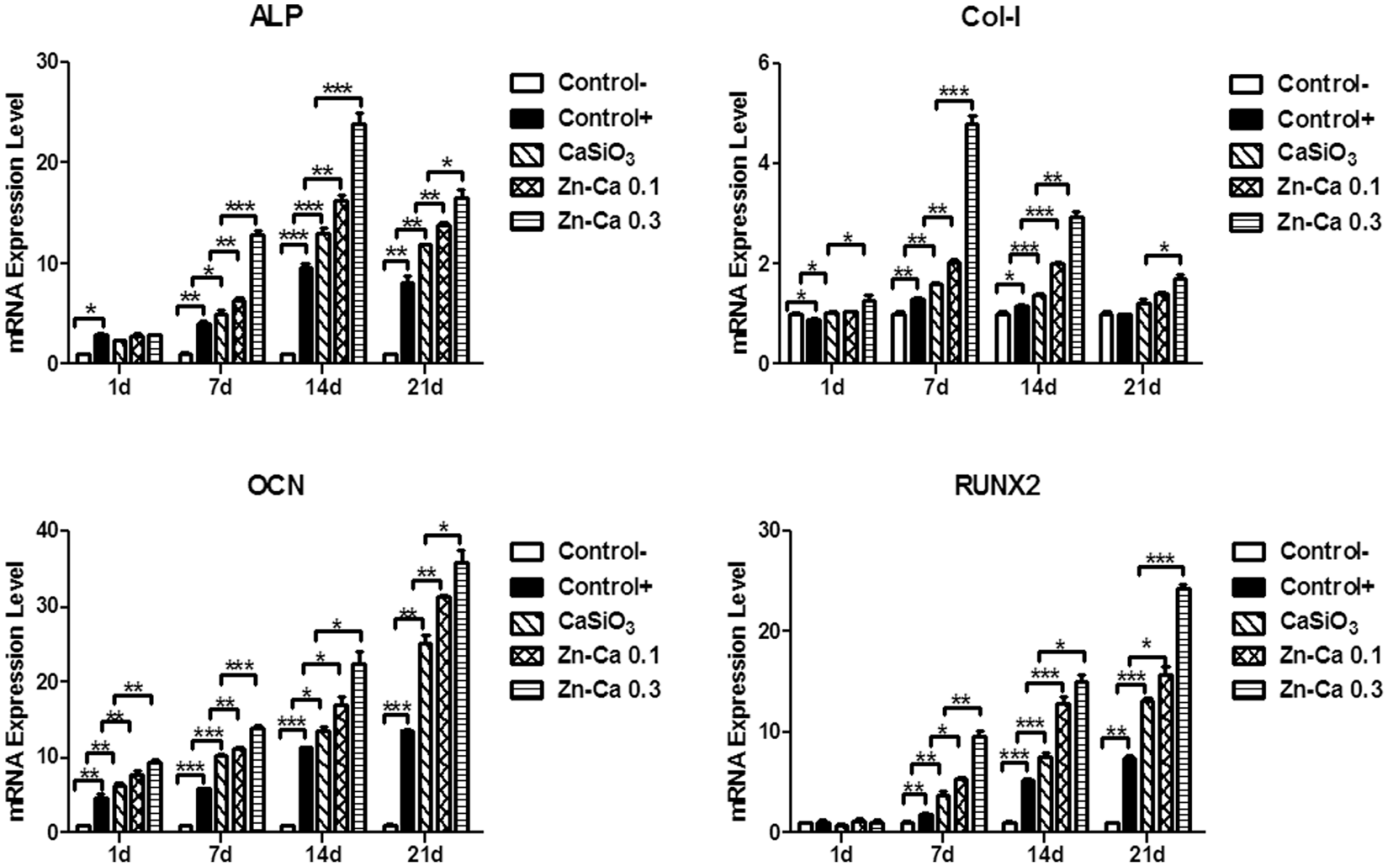
Quantitative real-time PCR analysis of the ALP, Col-I, OCN and RUNX-2 mRNA expression on different coatings at four time points (1, 7, 14 and 21 days). Data were calculated relative to the expression of the housekeeping gene GAPDH, which was used as the internal control. The results are presented as the mean ± SD for triplicate measurements. *p < 0.05, **p < 0.01, ***p < 0.001. Abbreviations: GAPDH, glyceraldehyde phosphate dehydrogenase; ALP, alkaline phosphatase; Col-I, procollagen α1(I); OCN, osteocalcin; RUNX-2, runt-related transcription factor 2. Negative control: BM-PCs on Ti-6Al-4V with growth medium. Positive control: BM-PCs on Ti-6Al-4V with osteogenic medium.

The expression of the specific osteogenic markers alkaline phosphatase (ALP), procollagen α1(I) (Col-I), osteocalcin (OCN) and runt-related transcription factor 2 (RUNX-2) was examined using quantitative polymerase chain reaction (Q-PCR). A detailed presentation of the fold change in the expression of these markers is shown in Fig. 2. For each experiment, BM-PCs were cultured on Ti-6Al-4V with the growth medium as a negative control, which had no detectable levels of the osteogenesis-specific genes. In addition, osteogenic differentiation was induced on Ti-6Al-4V exposed to osteogenic medium, which served as a positive control and expressed osteogenesis-related genes. The expression of an early stage osteoblast differentiation marker, ALP, was upregulated in BM-PCs cultured on the Zn-Ca 0.3 coating compared to cells cultured on the other coatings and the control, with maximum upregulation at day 14 for all treatment groups. However, at day 21, the ALP levels were lower than the mRNA expression observed on other days, and no significant difference was found at day 1. Col-I is also an early marker of osteogenic differentiation. The Col-I mRNA expression level decreased from day 7 for all samples, but the levels were significantly higher in cells seeded on the Zn-Ca 0.3 coating than those on the Zn-Ca 0.1 coating, the CaSiO_3_ coating and the control. The expression levels of the late osteogenic marker OCN increased in all groups with a time-dependent effect and significant upregulation for the Zn-Ca 0.3 coating compared with the Zn-Ca 0.1 coating, the CaSiO_3_ coating and the control at all time points. A similar trend was observed for the RUNX-2 mRNA expression.

### Zinc-modified Coatings Promote the Osteogenic Differentiation and Mineralization of BM-PCs

**Figure 3:**
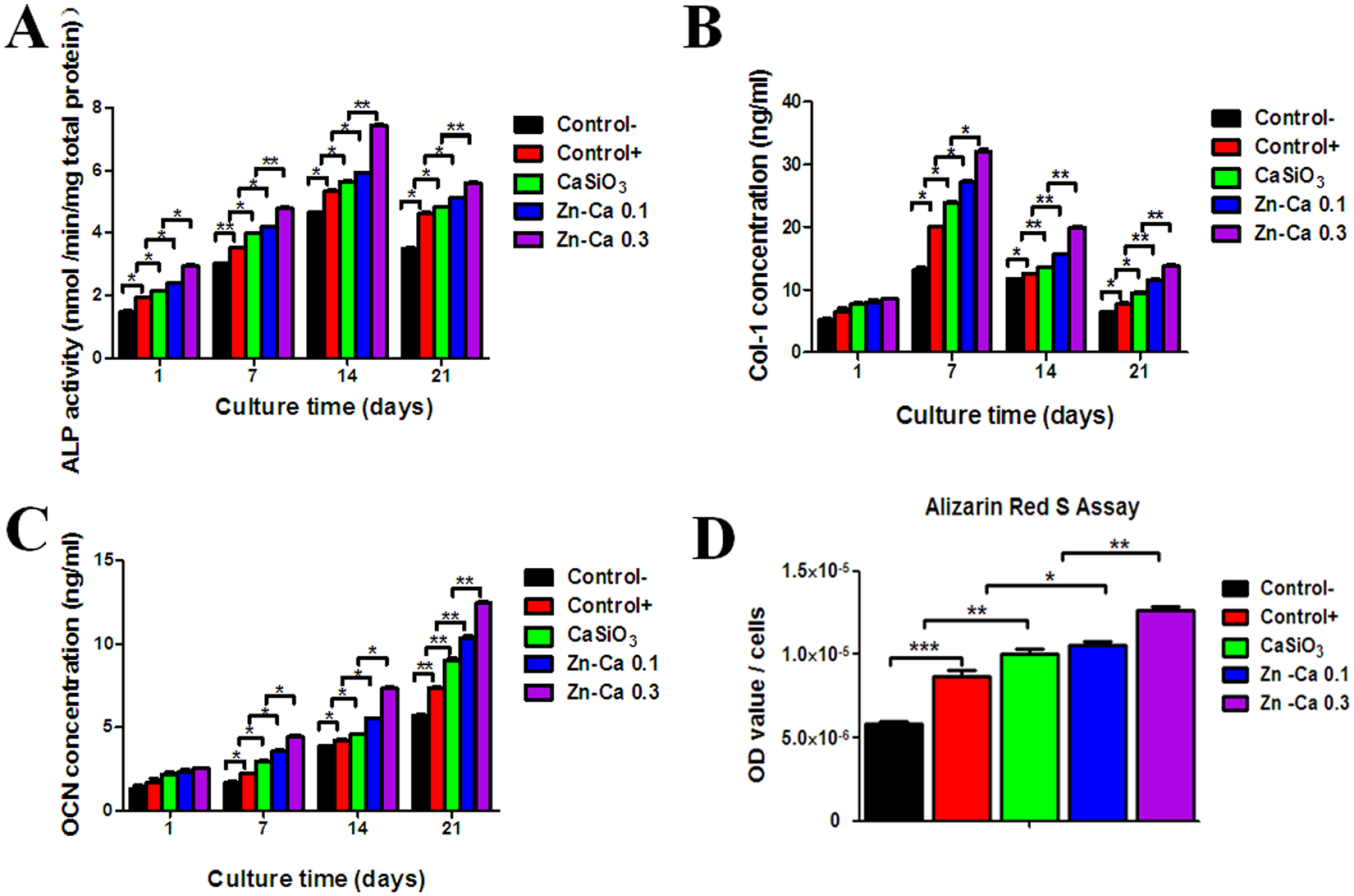
(**A**) ALP activity and (**B**) Col-I and (**C**) OCN levels of BM-PCs cultured on Ti-6Al-4V (control) and on the CaSiO_3_, Zn-Ca 0.1 and Zn-Ca 0.3 coating surfaces with or without the osteogenic medium at 1, 7, 14 and 21 days. (**D**) Semi-quantitative analysis of *in vitro* matrix deposition mineralization was performed at 21 days by Alizarin red S staining. Negative control: BM-PCs on Ti-6Al-4V with growth medium. Positive control: BM-PCs on Ti-6Al-4V with osteogenic medium. Each value represents the mean ± SD from triplicate determinations.*p < 0.05, **p < 0.01, ***p < 0.001.

BM-PCs cultured on various coatings were subjected to an ELISA assay. The ALP activity levels had a similar profile for all samples, with increased activity during the differentiation of BM-PCs into osteoblasts at all time points (Fig. 3A). The expression of ALP was upregulated to its highest levels after 14 days and then downregulated after 21 days in all samples. However, the enzymatic activity of ALP was significantly increased in BM-PCs cultured on the Zn-Ca 0.3 coating compared to the other groups.

Col-I levels were determined at 1, 7, 14 and 21 days of culture (Fig. 3B). The Col-I level decreased with time for all groups from 7 to 21 days, and no significant difference was found between all groups on the first day of culture. However, higher levels of Col-I were observed in cells cultured on the Zn-Ca 0.3 coating than in the other coating groups and the control group, and the level increased with the Zn/Ca ratio.

OCN was also used to evaluate the effect on the osteogenic differentiation of BM-PCs on different coatings *in vitro* (Fig. 3C). In the BM-PCs grown on all coatings, the OCN level increased with culture time. Moreover, cells on the Zn-Ca 0.3 coating at 21 days had significantly greater OCN levels than the other coating groups and the control group, although there was no significant difference in OCN levels in all groups at day 1. All the results showed striking similarities with the Q-PCR analysis, which suggested that the Zn-Ca 0.3 coatings have higher potential to induce the differentiation of BM-PCs into osteoblasts than all other groups.

To evaluate the osteogenic mineralization of the BM-PCs on the various coatings, Alizarin red S staining was employed to assess calcium deposition using a semi-quantitative analysis of cells at day 21 (Fig. 3D). The amount of calcium deposition was greatly increased in the Zn-Ca 0.3 coating group compared to all other groups. In addition, the level of mineralized deposition increased as the Zn-Ca ratio increased.

### Enhancement of the Osteogenic Differentiation of BM-PCs Cultured on Zn-modified Coatings through the TGF-β/Smad Signaling Pathway

**Figure 4:**
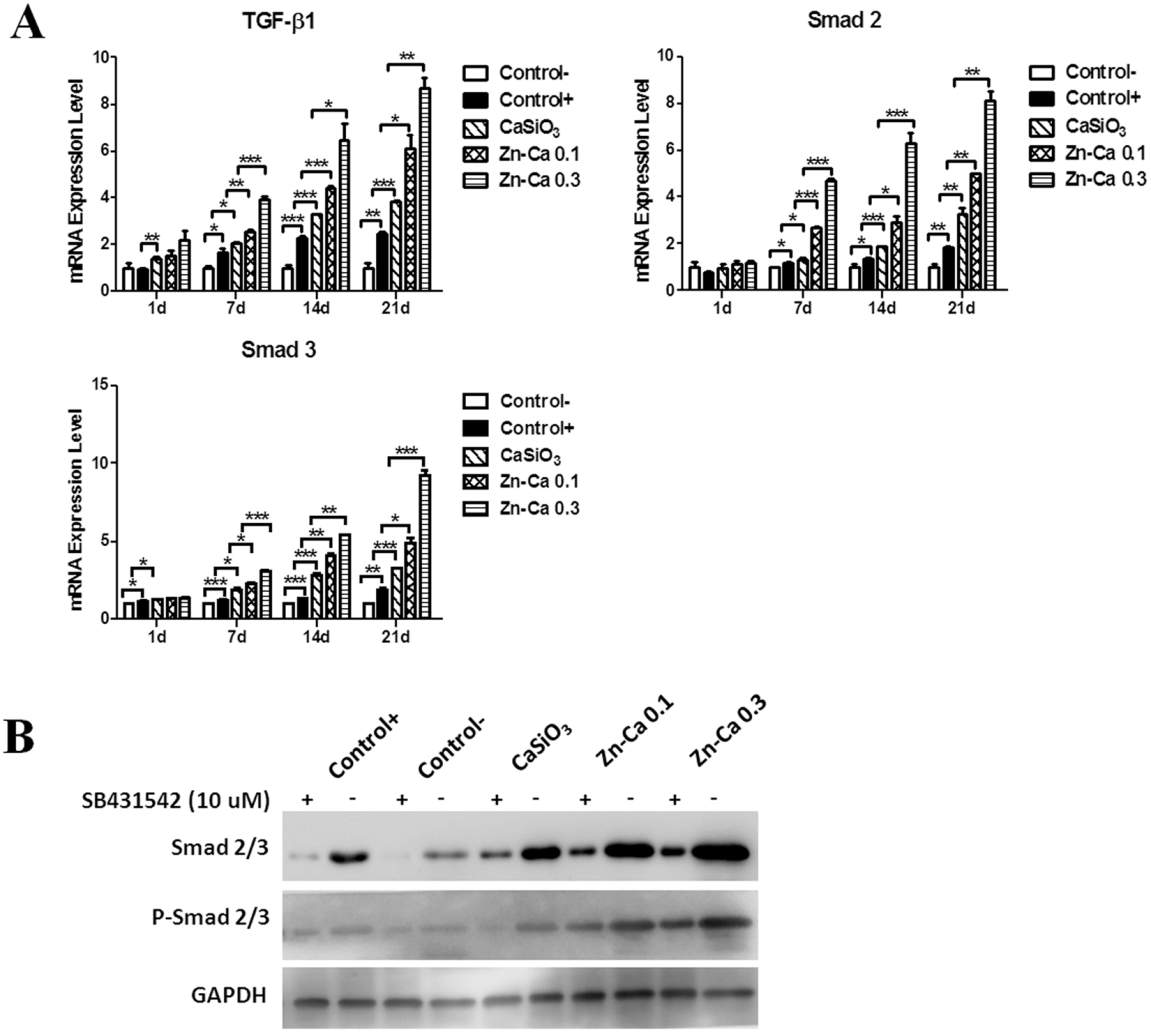
Detection of genes and proteins involved in the TGF-β/Smad signaling pathway. (**A**) Q-PCR quantification of genes involved in the TGF-β/Smad signaling pathway in BM-PCs cultured on Ti-6Al-4V (control) and on the CaSiO_3_, Zn-Ca 0.1 and Zn-Ca 0.3 coating surfaces at 1, 7, 14 and 21 days. The results were determined by the comparative CT method (2^−*ΔΔ*CT^). The bars represent the mean ± SD (n = 3). *p < 0.05, **p < 0.01, ***p < 0.001. (**B**) Western blot analysis of the expression of Smad2/3 and phosphorylated Smad2/3 in BM-PCs cultured on Ti-6Al-4V (control) and on the CaSiO_3_, Zn-Ca 0.1 and Zn-Ca 0.3 coatings for 21 days in the presence or absence of the ALK5 inhibitor SB431542 (10 μM). GAPDH served as an internal control. Negative control: BM-PCs on Ti-6Al-4V with growth medium. Positive control: BM-PCs on Ti-6Al-4V with osteogenic medium.

To understand the molecular mechanisms by which Zn-modified coatings promote osteogenic differentiation in BM-PCs, we first screened several signaling pathways associated with BM-PCs differentiation by Q-PCR. Several signaling pathways play significant roles in the regulation osteogenic differentiation on different surface coatings, including insulin-like factor-1 (IGF-1), mitogen-activated protein kinase (MAPK) and transforming growth factor-β1(TGF-β1) signaling^24–34^. In our study, the TGF-β/Smad signaling pathway played an essential role in the regulation of BM-PCs osteogenic differentiation. The Q-PCR results showed that the mRNA levels of TGF-β1, Smad2 and Smad3 all increased with incubation time for all coatings. Moreover, the associated genes were significantly increased in the Zn-Ca 0.3 coating compared with that of the other groups (Fig. 4A). However, no substantial enhancement with culture time was observed for the expression of genes in the classical MAPK signaling pathway, including insulin-like growth factor (IGF-I), extracellular signalregulated kinase1/2 (ERK 1/2) and protein kinase C-δ (PKC-δ), for any of the coatings (Figure S1). Therefore, our data indicated that Zn-modified coatings appeared to specifically and significantly activate the TGF-β/Smad signaling pathway during the osteogenic differentiation of BM-PCs.

The effect of Zn-modified coatings on TGF-β/Smad signaling pathway activation was further verified by Western blotting assays (Fig. 4B). Typically, receptor-regulated Smad (R-Smad) is activated by phosphorylation upon TGF-β/Smad signaling. The expression levels of key proteins involved in the TGF-β/Smad signaling pathway, including Smad2/3 and p-Smad2/3, were increased in the Zn-Ca 0.3 coating group compared to the other groups. In addition, SB431542, a highly selective inhibitor of Smad2/Smad3, was used. As expected, treatment with SB431542 decreased the Smad2/3 and p-Smad2/3 protein levels in a manner consistent with the effects of SB431542. This result suggests that the TGF-β/Smad signaling pathway plays a direct and significant role in regulating BM-PCs osteogenic differentiation on the different coatings, while neither the IGF-1pathway nor the MAPK pathway appear to be involved.

### Effects of Zinc-modified Coatings on Bone Regeneration *In Vivo*

#### Micro-CT Evaluation

**Figure 5:**
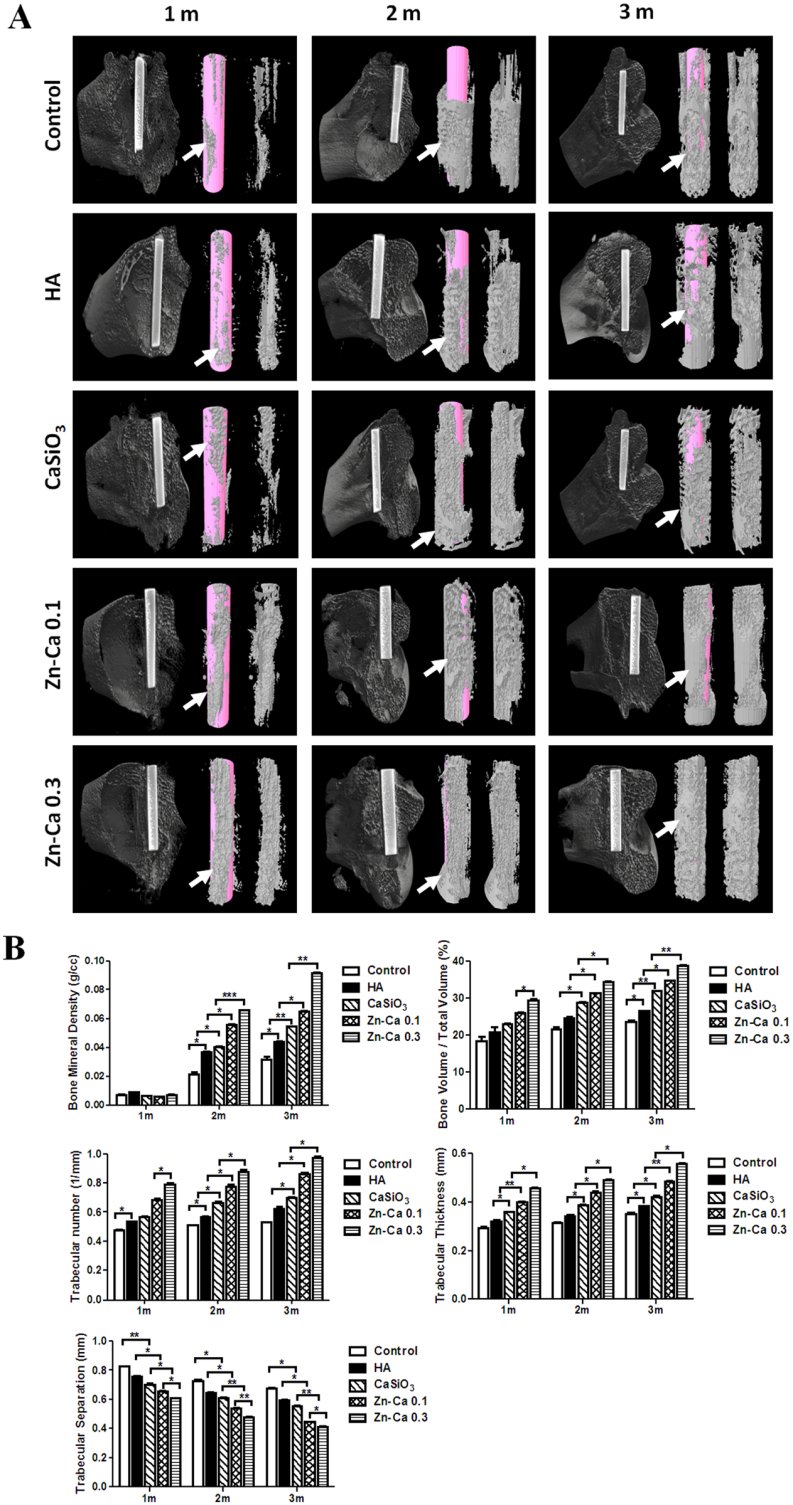
3-D micro-CT analysis for the assessment of newly formed bone tissue on the surface of Ti-6Al-4V and on hydroxyapatite (HA), CaSiO_3_, Zn-Ca 0.1 and Zn-Ca 0.3 coatings 1, 2, and 3 months after implantation in osteopenic rabbit femurs. (**A**) 3D images of trabecular bone (gray) on the surface of Ti-6Al-4V and HA, CaSiO_3_, Zn-Ca 0.1 and Zn-Ca 0.3 coatings (pink) at 1, 2, and 3 months post-implantation. White arrows indicate new bone formation. (**B**) Quantitative results of implant osseointegration and peri-implant microstructural parameters, such as bone mineral density, bone volume/total volume of bone, trabecular number, trabecular thickness and trabecular separation, on the surface obtained by micro-CT assessment 1, 2, and 3 months after implant insertion. All data are represented as the mean ± SD (n = 3). *p < 0.05, **p < 0.01, ***p < 0.001.

To investigate the effects of Zn-modified coatings on bone regeneration in osteopenic rabbits, bare (control) and hydroxyapatite (HA)-, CaSiO_3_-, Zn-Ca0.1- and Zn-Ca0.3-coated Ti-6Al-4V with a diameter of 2.0 mm and a length of 10 mm was implanted into the femur defects of osteopenic rabbits subjected to bilateral ovariectomy (OVX) in combination with methylprednisolone sodium succinate (MPS) (Figure S2). 3-D micro-CT was used to evaluate the differences in the bone-implant interface and trabecular microstructure of peri-implant bone tissue between the control and treated groups (Fig. 5A). At 1 month post-implantation, almost no new bone formation was apparent in the control or the HA and CaSiO_3_ coating groups, and a better performance of bone repair and integrity was found in the Zn-modified coating groups with increases in the Zn/Ca ratio. From 2 to 3 months post-implantation, all groups showed enhanced bone formation with implant time. 3D images indicated that the bone density on the surface of the Zn-Ca 0.3 coating was enhanced compared to that of the Ti-6Al-4V control and other coating groups. The osteogenic effect was in the order Zn-Ca 0.3 > Zn-Ca 0.1 > CaSiO_3_ > HA > control. This result suggests that the Zn-Ca 0.3 coating had the strongest promoting effect on bone formation with implant time.

The comprehensive quantitative analysis of all the micro CT parameters is shown in Fig. 5B. At 1 month post-implantation, the Zn-modified coating resulted in slight changes in all micro-CT parameters. From 2 to 3 months post-implantation, the HA, CaSiO_3_, Zn-Ca 0.1 and Zn-Ca 0.3 coating groups displayed a significant increase in all structural bone parameters, including bone mineral density (BMD), bone volume/tissue volume (BV/TV), trabecular number (Tb.N), and trabecular thickness (Tb.Th) and a lower trabecular separation (Tb.Sp) than the control. Moreover, the Zn-Ca 0.3 coating had the strongest effect on these parameters, and significant differences appeared within all five groups, which provided support for the results of the micro-CT images.

#### Histological Analysis

**Figure 6:**
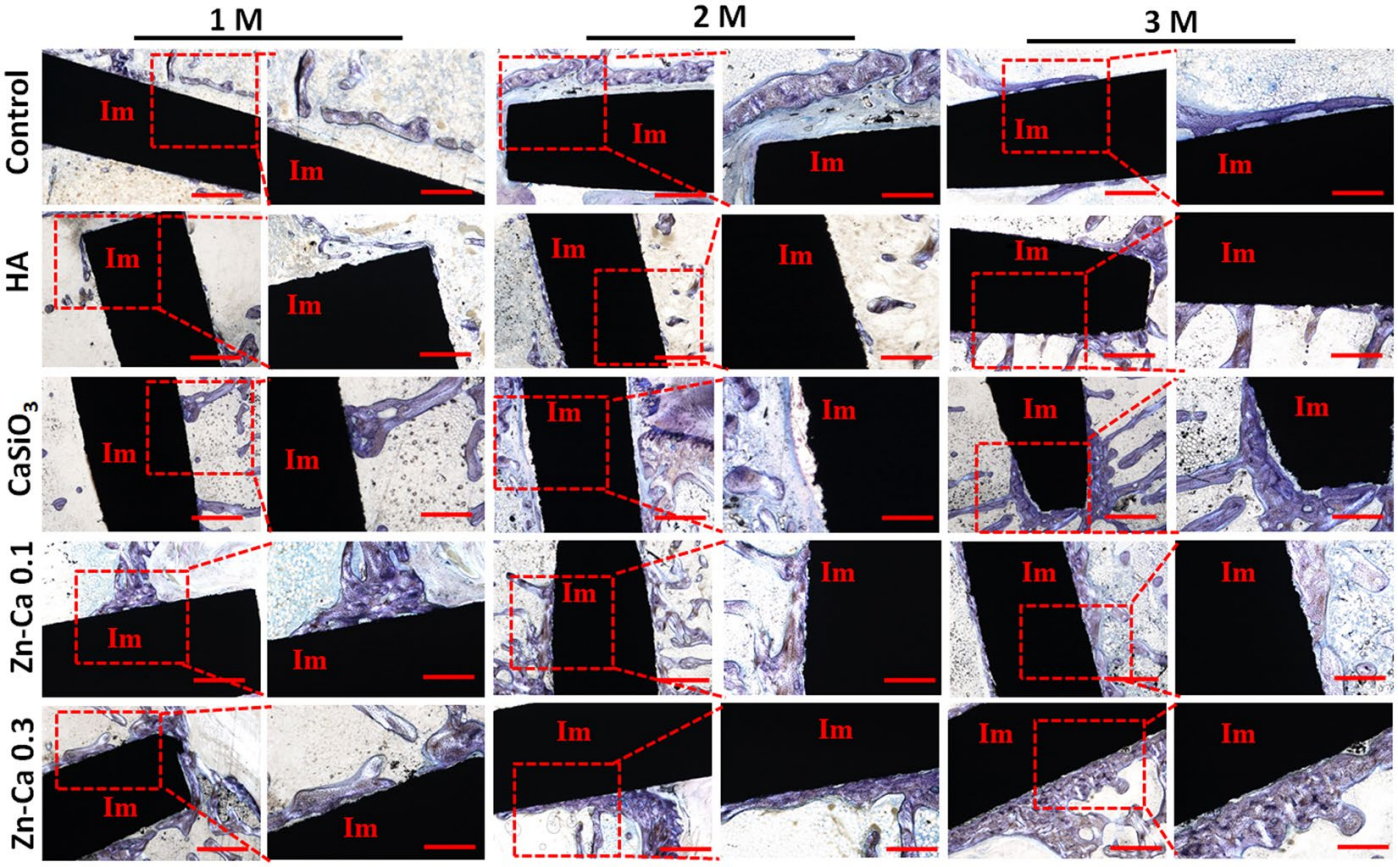
Histological, toluidine blue-stained sections from osteoporotic rabbits 1, 2, 3 months post-implantation for Ti-6Al-4V and the hydroxyapatite (HA), CaSiO_3_, Zn-Ca 0.1 and Zn-Ca 0.3 coatings. Right panel (Scale bar indicates 500 μm): magnification of the left red square in panel (Scale bar indicates 1000 μm) in each month. Im, implant; M, month.

The micro-CT analysis of the segmental defects was further supplemented by histological analysis. The photomicrographs of longitudinal sections of the bone defect sites of all coatings show the details of the bone-to-implant interface and peri-implant bone tissue (Fig. 6). The Ti-6Al-4V-implanted group did not form new bones along the integrated surfaces at 1 and 2 months post-implantation but did have a small amount of new bone formation away from the implant interface. Moreover, almost no osteointegration was observed around the implants at 3 months post-implantation. For the HA coating, a few new bones started to form at 1 month, more regenerated bone formed at some sites of the coating surfaces at 2 months, and limited osteointegration was found at 3 months post-implantation. Although new bone started to be induced in the CaSiO_3_ coating at 1 month, more new bones were regenerated along the integrated surfaces at 2 months, and a few showed osteointegration at 3 months. The Zn-Ca 0.1 and Zn-Ca 0.3 coating groups showed a prominent induction of new bone formation at 1 month, and a large amount of the new bone surrounding the implant was characterized by prominent osteoid seams at the surface of newly formed mineralized bone at 2, 3 months. In particular, we found that most of the new bone integrated with the coating surfaces in the Zn-Ca 0.3 coating group at 2 months, and all but the most recently formed bones were tightly integrated with the implantation surfaces at 3 months. Collectively, our results suggested that the Zn-Ca 0.3 coating improved osseointegration with the formation of a direct bone interface *in vivo*.

## Discussion

Calcium silicate (Ca-Si) ceramics have attracted great attention in bone tissue engineering because of their bioactivity and good osseointegration properties^35, 36^. However, there are still some drawbacks, including a relatively high dissolution rate in biological environments that affects long-term clinical performance especially in osteoporotic patients^37, 38^. Thus, various surface modification methods have been used to enhance the chemical stability and biological properties^39–43^. Some studies have suggested that incorporation of dopant Zn ions into bioactive coatings with appropriate concentrations positively affected the cellular responses^21, 44^. Higher zinc content could result in reduction in cell proliferation^45, 46^. In this study, we successfully prepared Zn-modified calcium silicate coatings with different Zn contents on Ti-6Al-4V by the plasma-spray method^47, 48^. We found that Zn modified titanium surface improved the osteogenic activity as the Zn/Ca mol ratio increased *in vitro* and *in vivo*.

Much research in bone tissue engineering has been devoted to adult stem cells. BM-PCs were used in the present study due to their high osteogenic capacity and clinical significance^49^. Our results showed that Zn-modified coatings significantly enhance proliferation in BM-PCs and are non-cytotoxic compared with CaSiO_3_ coatings and uncoated Ti-6Al-4V at all tested time points. Moreover, the cell proliferation rate correlated with the increase in the Zn/Ca mol ratio, consistent with our previous study and other studies reporting the stimulatory effect of zinc on osteoblastic cell behavior^41, 47, 50^. Therefore, the enhanced cell viability on the Zn-Ca 0.3 coating may be attributed to proper Zn release.

Previous research has demonstrated that osteoblast-specific factors might be important at several stages of bone regeneration^51, 52^. In the present study, the osteogenic markers ALP, Col-I, OCN and RUNX-2 were chosen to examine osteogenic differentiation *in vitro* by Q-PCR. Compared to the Zn-Ca 0.1 coating, the CaSiO_3_ coating and the control, the Zn-Ca 0.3 coating significantly upregulated osteogenesis-specific genes, such as ALP, Col-I, OCN and RUNX-2. ELISA analysis also indicated that the ALP activity and the Col-I and OCN secretion were stronger for BM-PCs on the Zn-Ca 0.3 coating than on the other coatings and the uncoated Ti-6Al-4V. Thus, notable differences in ALP, Col-I and OCN activity were related to the osteogenic effects of zinc. Similar results showing that various Zn concentrations have different effects on osteogenic differentiation were previously reported^53^. Furthermore, the effect of Zn-modified coatings on osteoblast differentiation was investigated by Alizarin red S staining. Bone nodules are osteoblastic phenotypic markers and represent the final stages of osteoblastic differentiation. Alizarin red S quantification revealed that the percentage of mineralized nodules increased with the Zn/Ca mol ratio and cells on the Zn-Ca 0.3 coating after 21 days had higher levels of calcium nodule formation than that in other groups. The results were in agreement with previous studies showing that Zn plays an important role in osteoblastic bone formation and mineralization and that Zn-containing implants could improve osteoblastic function through proper Zn-ion release^54, 55^. However, little is known about the mechanism underlying the Zn-modified coating promotion of osteogenic differentiation and bone regeneration.

**Figure 7:**
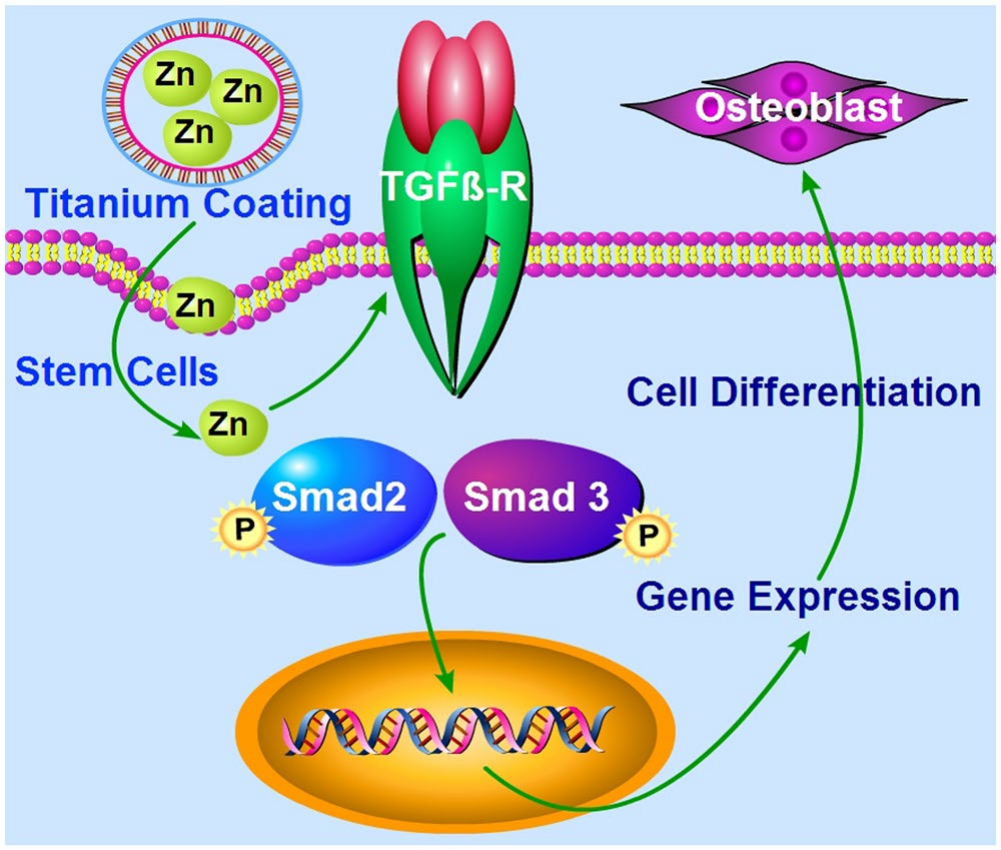
Plausible molecular mechanism of Zn-modified coatings modulating of osteogenic differentiation of BM-PCs through the TGF-β/Smad signaling pathway.

To further investigate the underlying molecular mechanisms, we searched for potential target genes associated with signaling pathways of BM-PCs osteogenic differentiation on the different coatings. Our results showed that Zn-modified coatings upregulated genes of the TGF-β/Smad signaling pathway, such as TGF-β1, Smad2 and Smad3. No change was observed with culture time for the expression of genes involved in the classical MAPK signaling pathway, including IGF-I, ERK 1/2 and PKC, in all the coatings, which suggested that Zn-modified coatings can activate the TGF-β signaling pathway and promote differentiation and mineralization in BM-PCs. The TGF-β pathway is important for BM-PCs differentiation into the osteogenic and chondrogenic lineages^56, 57^. TGF-β affects bone formation by activating their receptors to induce the phosphorylation of a group of intracellular transcription factors known as Smads^58^. *Smad2/3* serve as substrates for TGF-β receptors^59^. The activated Smad complex can then move into the nucleus to trigger the transcription of a set of target genes. In this study, the TGF-β inhibitor SB431542 was used to investigate the direct role of TGF-β signaling during the process of BM-PCs differentiation on the different coatings. Blocking TGF-β signaling resulted in reduced Smad2/3 and p-Smad2/3 protein levels, which indicated that the Smad2/3-mediated TGF-β signaling pathway is strongly involved in the Zn-modified coatings promoting differentiation and mineralization in BM-PCs (Fig. 7). In addition, Runt-related transcription factor 2 (RUNX-2) is the earliest osteogenic marker (*Runx*-*2*) that binds to the osteocalcin promoter^60–62^, interacts with Smads and subsequently induces the osteogenic marker genes ALP and Col-I at early stages and OCN at later stages of differentiation^63, 64^. Our results further support the fact that TGF-β signaling is an important event in BM-PCs proliferation and differentiation in a Smad-3-dependent manner^65^.

For biomedical implants, improving bone regeneration on implant surfaces is an important issue for the long-term success of the implant in both healthy and pathological conditions. The mains studies on biomaterial implants were performed in healthy animals^4, 5, 47^. However, the effects of titanium implants in the presence of an osteoporotic state are rarely reported. The most significant problem in treating osteoporotic fractures is unstable fixation arising from implant loosening because of the poor bone stock with low implant pull-out strength^66, 67^. The major contribution of this study is that shows that Zn-modified titanium improves implant osseointegration in ovariectomized rabbits as a model of osteoporosis. In *in vivo* study, the micro-CT results showed that Zn-modified coatings with higher Zn content can promote new bone formation to repair an osteoporosis metaphysis bone defect at early (1 month) and late time points (3 months). This function is correlated with the Zn content of the coatings and the implant time. Coatings containing higher amounts of Zn significantly increased BMD, BV/TV, Tb.N and Tb.Th and significantly decreased Tb.Sp compared to those in the other groups. Furthermore, the histomorphometric results also showed tighter contact with the surrounding host bone tissue with no obvious adverse effects and with accelerated new bone formation around the coating surface for the Zn-Ca 0.3 coating compared to the other coatings and pure titanium. These findings suggest that the Zn ions released from the Ca-Si-based coatings could influence the surrounding bone tissue, specifically by regulating the osteogenesis-related parameters of the environment for better integration of the titanium substrate with the host bone tissue, which was consistent with the previous reports that Zn has a positive effect on osteoblastic activity and bone formation^68, 69^. In other words, the Zn-modified coatings exhibited better properties for implant osseointegration in osteopenic rabbits and could have promising clinical potential for orthopedic implants, especially in the condition of osteoporosis.

## Conclusion

Zn-doped calcium silicate coatings with two different Zn contents were successfully prepared by plasma spraying. Zn-modified coatings significantly promoted the proliferation and osteogenic differentiation of BM-PCs *in vitro*, showing a remarkable increase in osteogenesis-specific gene markers, such as ALP, Col-I, OCN and RUNX-2, and higher levels of ALP activity, Col-I and OCN secretion and calcium deposition with obvious dose- and time-dependent tendencies. Moreover, we confirmed that Zn-modified coatings activated the TGF-β/Smad signaling pathway to regulate BM-PCs osteoblastic differentiation. Furthermore, the *in vivo* study demonstrated that Zn-modified coatings with a higher Zn content significantly enhanced bone contact and regeneration with host bone tissue in osteopenic rabbits. These findings suggested that Zn-modified coatings are good candidates with high potential for clinical applications for treating defective osteoporotic bone tissues in the future.

## Experimental Section

### Preparation and Characterization of Ca-Si-based Powders and Coatings

Zn-modified calcium silicate ceramic powders were synthesized using a previously described method^41, 47^. Briefly, zinc nitrate hexahydrate (Zn(NO_3_)_2_.6H_2_O; Sinopharm Chemical Reagent Co., Ltd.(SCRC), Shanghai, China), calcium nitrate tetrahydrate (Ca(NO_3_)_2_.4H_2_O; SCRC, Shanghai, China) and tetraethyl orthosilicate (TEOS, (C_2_H_5_O)_4_Si); SCRC, Shanghai, China) were used as raw materials. In the process, the mol ratio of Zn(NO_3_)_2_.6H_2_O:Ca(NO_3_)_2_.4H_2_O:C_2_H_5_O)_4_Si was x:1:1,with values of x = 0.1 and 0.3 (denoted by Zn-Ca 0.1 and Zn-Ca 0.3, respectively). An atmospheric plasma-spraying (APS) system (F4-MB, Sulzer Metco, Switzerland) was used to fabricate the coatings on Ti-6Al-4 V (Shanghai Yantai Metallic Material Co., Ltd., China) substrate with dimensions of ø 10 × 1 mm and ø 10 × 2 mm for the *in vitro* and *in vivo* study, respectively. The thickness of the coating was approximately 170 μm. CaSiO_3_ coatings were prepared using the same conditions as the control. Samples were ultrasonically cleaned and sterilized in acetone, ethanol and distilled water for 10 min.

### Isolation, Culture, and Identification of BM-PCs

All experiments involving animals complied fully with guidelines for animal care and use and the experimental protocol was ethically reviewed and approved by the Institutional Animal Care and Use Committee of The Second Military Medical University. The BM-PCs were isolated according to previously described methods^70^. Briefly, thigh bones were isolated from Sprague-Dawley rats (body weight, 100 ± 10 g), and bone marrow containing mononuclear cells was flushed out with Dulbecco’s modified Eagle medium: Nutrient Mixture F-12 (DMEM/F-12, Gibco Life Technologies, Carlsbad, CA, USA) using a 1 ml syringe. The cell suspension was filtered through a 40-μm strainer, and the cells were centrifuged (1000 rpm for 5 min) and washed with phosphate-buffered saline (PBS). The cells were resuspended in DMEM/F12 containing 10% fetal bovine serum (FBS; Gibco Life Technologies), 100 U/ml penicillin and 100 μg/ml streptomycin (Gibco Life Technologies). The harvested cells were seeded at a density of 1 × 10^6^/ml in a 75-cm^2^ tissue culture flask. The media were changed every 2–3 days. After 7 days of primary culture, the cells were passaged and expanded for future use. Cells at passages 3–5 were used in this study.

For BM-PCs characterization, cell surface markers were quantified by flow cytometry as previously described^71, 72^. BM-PCs of passage 4 were harvested by treatment with 0.25% trypsin-EDTA (Gibco Life Technologies). The trypsinized cells (1 × 10^6^ cells) were washed twice with PBS and stained with fluorescein isothiocyanate (FITC)-conjugated mouse antihuman CD29 and CD31, phycoerythrin (PE)-conjugated mouse antihuman CD90 and allophycocyanin (APC)-conjugated CD45 (BD Biosciences, San Jose, USA). After 30 min of incubation at room temperature, the cells were washed twice with PBS and analyzed using a BD FACS Aria II (BD Biosciences, San Jose, USA).

### Cell Proliferation and Cytotoxicity

Cell proliferation was measured using a Cell Counting Kit-8 (CCK-8; Beyotime, China). BM-PCs were cultured on the Ti-6Al-4V control and the CaSiO_3_, Zn-Ca 0.1, and Zn-Ca 0.3 coatings placed individually in a 24-well culture plate at a density of 1 × 10^4^ cells/cm^2^ in growth medium. After 1, 4, 7 and 14 days, the CCK-8 stock solution (10% of the total volume) was added to each well of the 24-well plates and incubated for 4 h at 37 °C and 5% CO_2_. Then, 100 μl of the solution was transferred to 96-well plates, and the absorbance was read at 450 nm by a microplate reader (SPECTRA MAX PLUS 384 MK3, Thermo Fisher Scientific, USA). Additionally, cell cytotoxicity was confirmed by a live/dead assay kit (Invitrogen, Carlsbad, CA, USA) after culturing for 48 h. The assay was performed according to the manufacturer’s instructions. Afterward, the stained cells were visualized using confocal laser scanning microscopy (CLSM, LeicaTCSSP5, Germany).

### Osteogenic Differentiation and Mineralization

To evaluate their osteogenic capacity, BM-PCs (1 × 10^5^ cells/well) were seeded on each coating group and cultured in osteogenic medium of DMEM/F-12 supplemented with 10% FBS, 100 U/ml penicillin, 100 mg/ml streptomycin, 50 µg/ml ascorbic acid (Sigma-Aldrich, St. Louis, USA), 1 μM dexamethasone (Sigma-Aldrich) and 10 mM β-glycerophosphate (Sigma-Aldrich). Cell differentiation and mineralization were confirmed using the alkaline phosphatase (ALP) activity, the procollagen α1(I) (Col-I) and osteocalcin (OCN) secretion and Alizarin red S staining as described below.

### ALP Assay and Col-I and OCN Secretion

The stable *p*-nitrophenol phosphate substrate was used to quantify ALP activity. BM-PCs were cultured on the Ti-6Al-4V control and the CaSiO_3_, Zn-Ca 0.1, and Zn-Ca0.3 coatings in osteogenic medium for 1, 7, 14, and 21 days. At each time point, the culture medium was removed, and the cells were washed with PBS and harvested in 1 ml of universal ALP buffer (100 mM citric acid, 100 mM KH_2_PO_4_, 100 mM sodium tetraborate.10H_2_O, 100 mM Tris, and 100 mM KCl; pH 11). The cells were centrifuged at 3000 rpm for 5 min at 4 °C. The ALP activity in the supernatants was determined following the addition of *p*-nitrophenyl phosphate substrate, and the reaction was stopped using 100 µl of 0.1 N NaOH. The absorbance was read with a microplate reader (SPECTRA MAX PLUS 384 MK3, Thermo, USA) at a wavelength of 405 nm.

The levels of Col-I and OCN secreted into the culture medium were quantified using an enzyme-linked immunoassay (ELISA) kit (R&D Systems, Minneapolis, MN, USA) following the manufacturer’s instructions.

### Alizarin Red Staining (ARS)

Osteogenesis was confirmed by Alizarin red S staining. The cells were seeded on different coatings and cultured in osteogenic medium. At the indicated time point, the cells were fixed with 4% paraformaldehyde for 20 min at room temperature and stained with 0.1% Alizarin Red S (Beyotime, China) at room temperature for 30 min. Afterward, the cells were washed twice with PBS and air dried before the ARS staining was eluted with 5% perchloric acid (SCRC, Shanghai, China). The 100 μl solution from each well was then transferred to a 96-well plate, and the optical density (OD) was measured at 490 nm using a spectrophotometer (SPECTRA MAX PLUS 384 MK3, Thermo, USA).

### RNA Extraction and Quantitative Reverse Transcriptase Polymerase Chain Reaction (Q-PCR)

After seeding for 1, 7, 14 and 21 days, total RNA was extracted from BM-PCs on each coating using RNAiso Plus (TaKaRa Bio Inc., Japan) and treated with Recombinant DNase I (RNase-free) (TaKaRa Bio Inc., Japan) to remove genomic DNA contamination following the manufacturer’s protocol. 500 ng RNA was converted to cDNA with M-MLV reverse Transcriptase (Promega, WI, USA) according to the manufacturer’s instructions. The PCR reaction with a final volume of 20 µl contained 10 µl of 2× SYBR Green PCR Master Mix, 1 µl of 5 µM forward and reverse primers, 8 µl of water, and 1 µl of template cDNA. The PCR amplification conditions were as follows: 95 °C for 5 min, 40 repetitions of 95 °C for 30 s, 60 °C for 30 s, and 72 °C for 30 s and a final extension at 72 °C for 10 min. The specific primers used for the qPCR are listed in the Supporting Information (Table S1). Q-PCR analysis was performed on an ABI 7500 Real-Time PCR System (Applied Biosystems, Life Technologies, USA). The expression of all target genes was normalized to the housekeeping gene glyceraldehyde 3-phosphate dehydrogenase (GAPDH) and shown as the expression relative to that of the negative control group using the comparative CT method (2^−*ΔΔ*CT^). Each experiment was repeated in triplicate for each individual sample.

### Western Blotting Analysis

A Western blot can be used to determine the specific protein expression in a given sample. BM-PCs in each group were lysed in RIPA buffer containing 1 mM phenylmethane sulfonyl-fluoride (Beyotime, China). The total protein concentration of the supernatant was measured using a bicinchoninic acid (BCA) assay kit (Beijing CoWin Biotech Co. Ltd., Beijing, China) in accordance with the manufacturer’s protocol. 10 μg protein from each sample were separated by sodium dodecyl sulfate-polyacrylamide gel electrophoresis (SDS-PAGE) and transferred onto 0.22 μm polyvinylidene difluoridemembranes (Millipore, Bedford, Mass, USA). After the membrane was blocked, the blots were incubated with the appropriate primary antibodies: rabbit anti-phospho-Smad2 (Ser465/467)/Smad3 (Ser423/425) (Cell Signaling Technology, Danvers, USA), rabbit anti-Smad2/3 (Cell Signaling Technology), and anti-GAPDH monoclonal antibody (Novus, CO, USA) overnight at 4 °C. All primary antibodies were applied at a 1:1000 dilution. The membranes were washed three times in phosphate-buffered saline +0.1% Tween 20 (PBST) buffer and then further incubated with anti mouse/rabbit HRP conjugated secondary antibodies at 1: 4000 dilutions for 1 h at room temperature and detected with the ECL Kit (Beijing CoWin Biotech Co. Ltd., Beijing, China). The protein bands were visualized using an Image Quant Las4000mini (GE Healthcare, UK). GAPDH served as an internal control.

### Inhibition of TGF-β/Smad Signaling

To assess the role of TGF-β/Smad signaling in the regulation of osteogenic differentiation of BM-PCs cultured on the Zn-modified coatings, on the second day of culture, BM-PCs on the different coatings were treated with or without 10 μM SB431542 (Sigma-Aldrich, St. Louis, USA). SB431542 is a selective inhibitor of activin receptor-like kinase ALK5 (TβRI), whereas Smad2 and Smad3 are substrates for ALK5. SB431542 has been demonstrated to be a specific inhibitor of the TGF-β/Smad pathway^73^. After 21 days, the BM-PCs were collected, and Smad2/3and phospho-Smad2/Smad3 proteins were detected by Western blotting.

### Ovariectomized Rabbits

Forty-five mature female New Zealand white rabbits (90–100 days old, 2–2.5 kg) were used for this experiment. The use of animals and the surgical procedures were approved by the Institutional Animal Welfare Committee of The Second Military Medical University. To induce an osteopenic model, the rabbits were subjected to bilateral ovariectomy (OVX) through a ventral incision under general anesthesia with an intraperitoneal injection of sodium pentobarbital (30 mg/kg body weight). The incision was closed layer by layer. After the surgery, the rabbits were injected intramuscularly with methylprednisolone sodium succinate (MPS) (Pfizer Manufacturing Belgium NV) dissolved in 0.9% benzyl alcohol at a dosage of 1.0 mg/kg/day for 4 consecutive months. Sham-operated rabbits were surgically operated on similarly to OVX rabbits except that the ovaries were not cauterized. At the end of the treatment, the osteopenic state in ovariectomized rabbits was confirmed by micro-computed tomography (micro-CT) imaging of the proximal tibia.

### Surgical Procedure

After osteopenic animal models were created, each ovariectomized rabbit underwent general anaesthesia by the intraperitoneal injection of sodium pentobarbital (30 mg/kg body weight). A linear skin incision that was approximately 2 cm long in the distal femoral epiphysis was made laterally, and the lateral femoral condyle was exposed by blunt dissection of the muscles. Then, circular holes (2-mm diameter, 10-mm depth) were created using a surgical electric drill at a slow speed. During drilling, physiological saline was supplied to remove bone shards and rinse the wounded area to stop the bleeding. Forty ovariectomized rabbits were randomly divided into five groups (8 rabbits per group), and the rabbits were implanted with (1) uncoated Ti-6Al-4V (control), (2) HA-coated Ti-6Al-4V, (3) CaSiO_3_-coated Ti-6Al-4V, (4) Ca_2_ZnSi_2_O_7_-coated Ti-6Al-4V (Zn-Ca 0.1) and (5) Ca_2_ZnSi_2_O_7_-coated Ti-6Al-4V (Zn-Ca 0.3). A total of four implants were implanted into the femur of each rabbit. Two implants were inserted into the femur of the left hind leg and another two into the femur of the right hind leg. When the implants were then gently placed to fill the grilled defects according to group allocation. Subsequently, the incision was closed with absorbable sutures (Marlin, Germany), and antibiotics and analgesics were injected intramuscularly. At each time point (1, 2 and 3 months after surgery), ovariectomized rabbits were euthanized and specimens were harvested for micro-computed tomography (micro-CT) analysis and histological examination.

### Micro-CT Examination

The femoral heads were fixed with 4% paraformaldehyde for 24 h at 4 °C. A micro-CT imaging system (SkyScan1076, Brukermicro CT, USA) was used to evaluate the new bone formation within the defect region. The acquisition settings were 49 kV and 200 μA with a spatial resolution of 35 μm. A consistent volume of interest (VOI) with a diameter of 3 mm and a height of 4 mm was chosen to evaluate the level of bone regeneration and was reconstructed three-dimensionally using Micview software. The bone mineral density (BMD), bone volume/total volume of bone (BV/TV), trabecular number (Tb.N), trabecular thickness (Tb.Th), and trabecular separation (Tb.Sp) were automatically determined to evaluate the new bone formation within the defect region of the femoral heads. All the morphometric analysis was performed using SkyScan CTVOX 2.1.

### Histopathological Examination

After micro-CT scanning, the defective femoral tissue was fixed in 4% paraformaldehyde for 3 days, dehydrated in a series of graded concentrations of ethanol from 70 to 100% for 1 day at each concentration, exposed to dimethyl benzene and then embedded in methyl methacrylate without decalcification., Each specimen was then cut perpendicular to the long axis of implants into 3 μm thick sections along the central axis of the implant using a cutting machine (EXAKT 300 CPB and System, Norderstedt, Germany). The sections from each group were stained with 1% toluidine blue and observed by light microscopy (BX51; Olympus, Japan) for histomorphometry.

### Statistical Analysis

For statistical analysis, Levene’s test was performed to determine the homogeneity of variance for all data, and then one-way analysis of variance followed by Tukey’s or Tamhane’s T2 posthoc test for multiple comparison was performed for the comparisons between different groups. All statistical analysis was performed using GraphPad Prism 5.0 (San Diego, CA, USA). The results were considered significant at *p < 0.05, very significant at **p < 0.01 and extremely significant at ***p < 0.001. All data were expressed as the mean ± standard deviation (SD).

## Electronic supplementary material

Supporting information
