# Supplementary material for: Zinc-modified Calcium Silicate Coatings Promote Osteogenic Differentiation through TGF-β/Smad Pathway and Osseointegration in Osteopenic Rabbits
Source: Sci Rep. 2017 Jun 13;7:3440. doi: 10.1038/s41598-017-03661-5 (PMC5469779; doi:10.1038/s41598-017-03661-5)
Supplement: Supplementary file 1 — Supporting information [file 41598_2017_3661_MOESM1_ESM.pdf]

Supporting Information

Zinc-modified Calcium Silicate Coatings Promote Osteogenic Differentiation through TGF-β/Smad Pathway and Osseointegration in Osteopenic Rabbits

Jiangming Yu[1,4]*, Lizhang Xu[1], Kai Li[2], Ning Xie[1], Yanhai Xi[1], Yang Wang[1], Xuebin Zheng[2], Xiongsheng Chen[1], Meiyan Wang[3]*and Xiaojian Ye[1]*

1. Department of Orthopaedics, Changzheng Hospital of Second Military Medical University, Shanghai, 200003, China

2. Key Laboratory of Inorganic Coating Materials, Shanghai Institute of Ceramics, Chinese Academy of Sciences, Shanghai, 200050, China

3. Shanghai Key Laboratory of Regulatory Biology, Institute of Biomedical Sciences and School of Life Sciences, East China Normal University, Shanghai, 200241, China

4. National Engineering Research Center for Nanotechnology, Shanghai, 200241, China

Jiangming Yu, Lizhang Xu and Kai Li contributed equally.

Correspondence authors: Jiangming Yu*, Email: yjm_st@163.com; Meiyan Wang*, Email: 061022049@fudan.edu.cn; Xiaojian Ye*, Email: yespine@163.com.

1. Results and Discussion

1.1 Characterization of BM-PCs

BM-PCs surface marker analysis by flow cytometry revealed that these fibroblast-like cells expressed high levels of the BM-MSCs markers CD 29 and CD 90, rarely expressed CD 31 and CD 45 (**Figure S3**), indicating high purity of BM-PCs were obtained.

1.2 Assessment of ovariectomized (OVX) rabbits model

To investigate whether Zn-modified coating can enhance bone regeneration in challenging diseased state, ovariectomized (OVX) rabbits as a model of osteopenia were used in our study. To confirm osteopenic rabbits, micro-CT was performed 4 months after ovariectomy (**Figure S2**). The effects of OVX on their skeletons are manifest, which bone loss in OVX rabbits are more than sham-operated rabbits. Moreover, bone mineral density (BMD), bone volume /tissue volume (BV/TV) after OVX calculated by Micro-CT were significantly reduced compared to that in sham-operated rabbits. These results indicated the successful establishment of osteopenia in OVX rabbits.

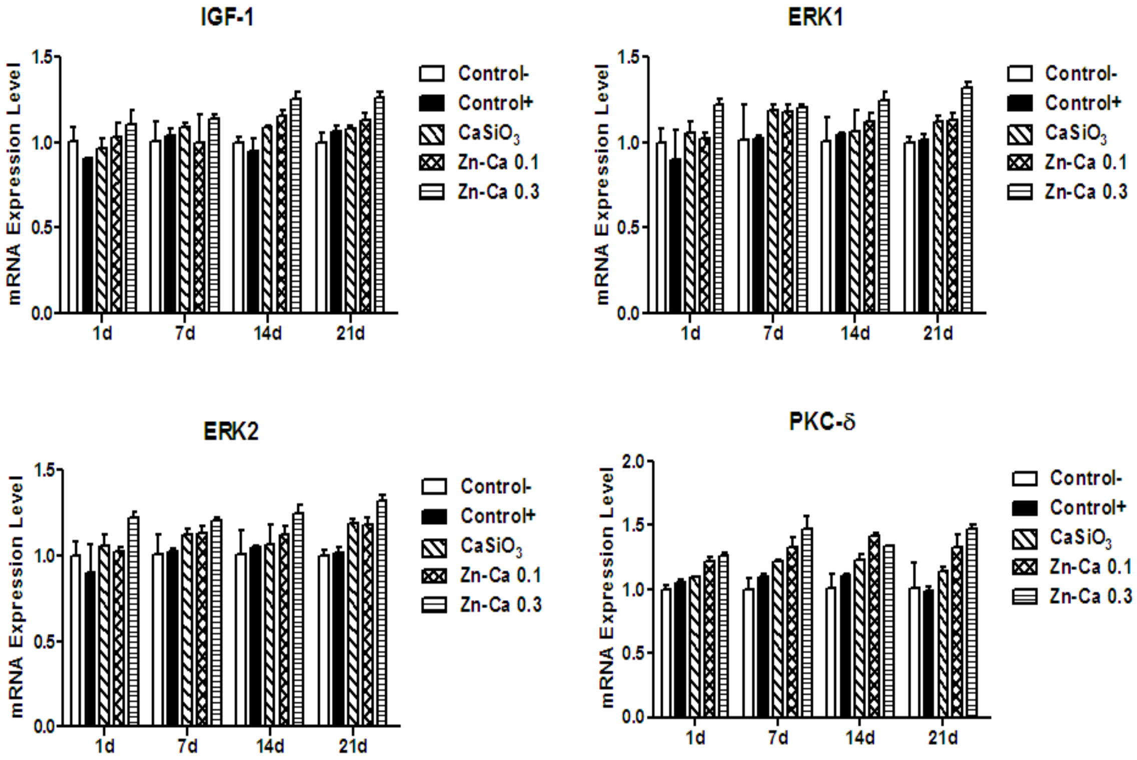

Figure S1 Genes expression associated with the classical MAPK signaling pathway, such as IGF-I, ERK 1/2, PKC-δ, in BM-PCs cultured on the Ti-6Al-4V (control) and the $CaSiO_3$, Zn-Ca 0.1 and Zn-Ca 0.3 coating surfaces at 1, 7, 14 and 21days were analyzed by Q-PCR. Data were calculated relative to the expression of the housekeeping gene GAPDH using the comparative CT Method ($2^{-\Delta\Delta CT}$). Results represent the mean ± SD (n = 3). Abbreviations: GAPDH, glyceraldehyde phosphate dehydrogenase; IGF-I, insulin-like growth factors; ERK 1/2, extracellular signalregulated kinase1/2 ; PKC-δ, protein kinase C-δ. Negative control: BM-PCs on Ti-6Al-4V with growth medium. Positive control: BM-PCs on Ti-6Al-4V with osteogenic medium.

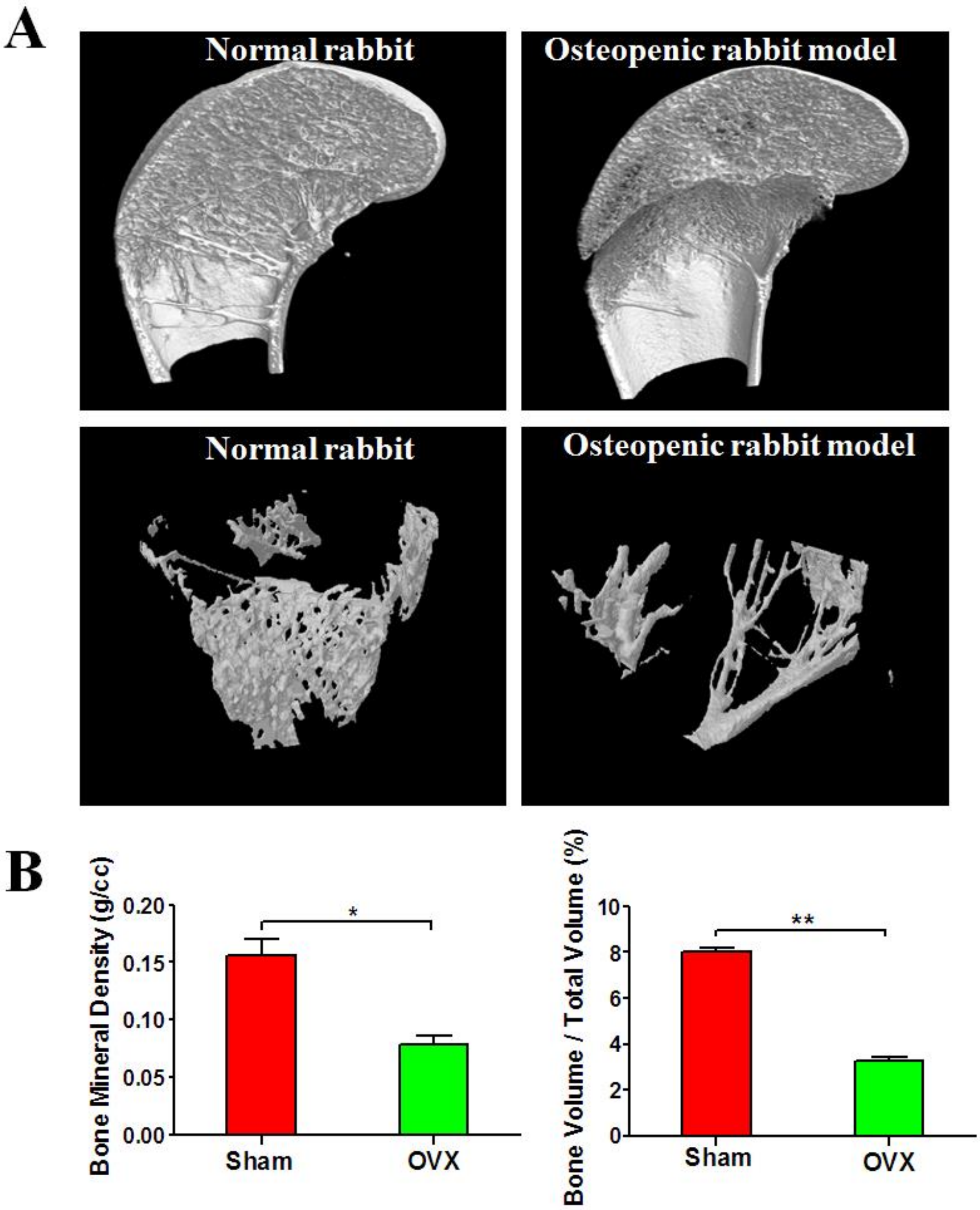

Figure S2 Verification of osteopenic state in ovariectomized rabbits. (A) 3D micro-CT images of proximal tibia at 4 months for ovariectomized (OVX) rabbits and sham-operated rabbits from axial section and cross section. (B) The comparison of the microstructural parameters, bone mineral density (BMD) and bone volume /tissue volume (BV/TV) of bone in the ovariectomized model group 4 months after ovariectomy were significantly lower than sham-operated group. Data presented as mean ± SD (n = 3). * $p < 0.05$, ** $p < 0.01$.

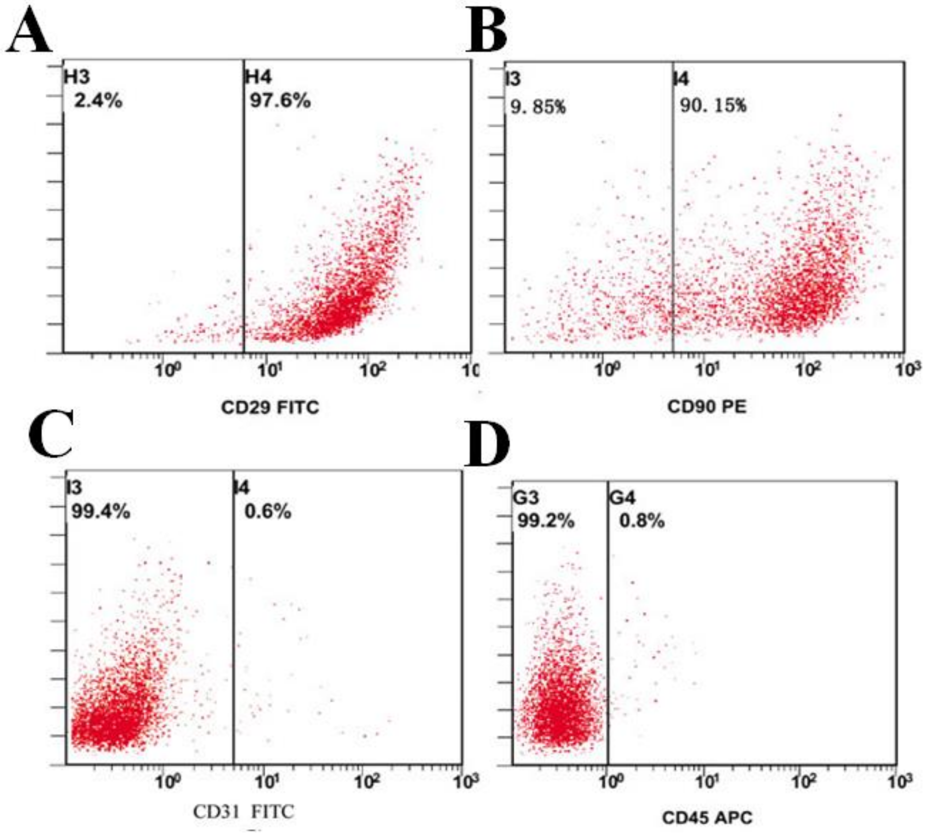

Figure S3 Characterization of rat BM-PCs using flow cytometry. BM-PCs expressed CD 29 (A) and CD 90 (B), whereas rare cells expressed CD31 (C) and CD45 (D).

Table S1 The sequences for primers of Q-PCR

Gene name	Primer sequence	Product length (bp)	Annealing temperature (℃)
ALP	5'-TGGTACTCGGACAATGAGATGC-3'<br>5'-GCTCTTCCAAATGCTGATGAGGT-3'	219	60
Col -I	5'-TTCTCCTGGTAAAGATGGTGC-3'<br>5'-GGACCAGCATCACCTTTAACA-3'	255	60
OCN	5'-AGGGCAGTAAGGTGGTGAATAGA-3'<br>5'-GAAGCCAATGTGGTCCGCTA-3'	146	60
RUNX-2	5'-AGTGGACGAGGCAAGAGTTTC-3'<br>5'-CCTTCTGGGTTCCCGAGGT -3'	205	60
TGF-β1	5'-GACCGCAACAACGCAATCTAT-3'<br>5'-ACCAAGGTAACGCCAGGAAT-3'	207	60
Smad2	5'-ACCCGAATGTGCACCATAAGAA-3'<br>5'-GCGAGTCTTTGATGGGTTTAGGA-3'	198	60
Smad3	5'-GTCAACAAGTGGTGGGGTGTG-3'<br>5'-GCAGGAAAGGCTTCTGGGATAA-3'	150	60
IGF-I	5'-CACTCATCCACAATGCCTGTCT-3'<br>5'-CTGAGCTGGTGGATGCTCTTC-3'	118	59
ERK1	5'-CCAGAGTGGCTATCAAGAAG-3'	181	59

	5'-TCCATGAGGTCCTGAACAA-3'		
ERK2	5'-TGCCGTGGAACAGGTTGT-3'<br>5'-TGGGCTCATCACTTGGGT-3'	153	59
PKC-δ	5'-GAGGCACTCACCACAGAC-3'<br>5'-AGG TCC AGC CAG AAC TCA-3'	204	60
GAPDH	5'-TTCGACAGTCAGCCGCATCTT-3'<br>5'- ATCCGTTGACTCCGACCTTCA-3'	281	60

Shown are the details of the primers used for Q-PCR, including melting temperatures, forward and reverse sequences and product length. ALP, alkaline phosphatase; Col-I, procollagen 1(I); OCN, osteocalcin; RUNX-2, runt-related transcription factor 2; TGF-β1, transforming growth factor-β1; Smad2, Sma and MAD-related Protein 2; Smad3, Sma and MAD-related Protein 3; IGF-1,insulin like factor-1; ERK 1, extracellular signalregulated kinase 1; ERK 2, extracellular signalregulated kinase 2; PKC-δ, protein kinase C-δ; GAPDH, glyceraldehyde-3-phosphate dehydrogenase.
